# Organic Acids and Polyphenols Determination in Polish Wines by Ultrasound-Assisted Solvent Extraction of Porous Membrane-Packed Liquid Samples

**DOI:** 10.3390/molecules24234376

**Published:** 2019-11-29

**Authors:** Alicia D. Robles, Magdalena Fabjanowicz, Justyna Płotka-Wasylka, Piotr Konieczka

**Affiliations:** 1 Department of Chemistry, Bromatology, Faculty of Exact and Natural Sciences, National University of Mar del Plata, 3350 Funes Street, Mar del Plata, Buenos Aires 7600, Argentina; aliciadrobles@gmail.com; 2 Department of Analytical Chemistry, Faculty of Chemistry, Gdańsk University of Technology, 11/12 Narutowicza Street, 80-233 Gdańsk, Poland; piotr.konieczka@pg.edu.pl

**Keywords:** wine, organic acids, polyphenols, UAPM-LS, GC–MS, Eco-Scale, GAPI

## Abstract

In the near future, Poland is going to have more and more favorable conditions for viticulture. Organic acids and polyphenols are among the most commonly analyzed compounds due to their beneficial properties for human health and their importance in the winemaking process. In this work, a new technique involving ultrasound-assisted solvent extraction of porous membrane-packed liquid samples (UASE-PMLS) was for the first time described and applied for real samples. The methodology based on UASE-PMLS for organic acids and polyphenols in wine samples was optimized and validated. Using the new technique coupled to GC–MS, organic acids and polyphenols were evaluated in Polish wine samples. Extraction solvent, extraction temperature, derivatization time and sample pH were optimized. Chemometric tools were used for data treatment. Good linearity was obtained for the concentration ranges evaluated with *r* values between 0.9852 and 0.9993. All parameters of method validation (intra- and inter-day precision and matrix effect) were over 80% with coefficient of variation (CV) up to 17%. Recovery was between (92.0 ± 8.5)% and (113 ± 16)%. Finally, green assessment was evaluated using Analytical Eco-Scale and Green Analytical Procedure Index (GAPI). The UASE-PMLS is characterized by many advantages, e.g., the extraction process is fast and easy coupled to GC–MS. Regarding other extraction techniques, the amount of used solvent is minimum, and no waste is generated. Therefore, it is an environmentally friendly technique.

## 1. Introduction

Nowadays, there is an increasing interest in grapevines and winemaking technologies in Poland. This is influenced by appearance of new vine assortments composed of hybrid varieties, which are suitable for the Polish climate. As the climate is changing due to global warming, and Poland is going to have more and more favorable conditions for viticulture, winery started to be perceived as a new source of income in Polish agriculture. Furthermore, there is an increased consumer awareness regarding the dietetic and healthy potential properties of wine, which also significantly influence the increased interest in wine [1]. Polish wines have their own specific characteristics. Due to their geographical location they are described as cold-climate wines, which give the perception of delicacy and refinement when consumed. Greater acidity gives freshness to this type of wine [1].

The composition of wine is characterized, mainly, by the content of a complex mixture of compounds at varietal concentrations. Organic acids are important for the wine stability and for their contribution to the organoleptic characteristics (flavor, color, and aroma) of wines. Organic acids are strongly connected to the aroma and taste of wine [2]. Therefore, their analysis in wines is required for the quality control as well as to check the evolution of acidity during the different stages of winemaking (starting from grape juices, continuing to the alcoholic fermentation, malolactic fermentation (depending of the style of wine), and wine stabilization processes), since important changes in wine can be detected by alterations in the acid content. During the malolactic fermentation, lactic acid and carbon dioxide are formed, due to the bioconversion of malic acid. This process improves the biological stability of the red wine and prevents the malic acid from being used by other microorganisms in bottled wine [3].

Regarding the bioactive capacity of organic acids, little is known about their beneficial effects on human health. Moreover, there is a lack of knowledge regarding the beneficial effects of the consumption of foods rich in these compounds, with the exception of ascorbic acid, which has a high antioxidant power [4]. However, there are more and more studies examining the organic acids’ characteristics, searching for positive effects of given compounds on the human body [5,6,7].

Polyphenols are one of the most abundant group of compounds present in grapes and thus in wines. Some of them, known as anthocyanins, are coloring substances. They are responsible for the red and blue color of red grapes and transfer their dark color to the red wine. In addition, tannins give soft astringency or roughness to the taste. This sensation can be perceived when chewing grape skins and drinking red wines, due to tannins passing from the skins to the wine during the winemaking process. In addition, there are also polyphenols that provide aromas and flavors to red wines [8]. White wines typically do not have polyphenols present in such large quantities as red wine, and the antioxidant activity is also subsequently lower. This is due to the fact that white wines are usually made from the free running juice, without grape mash, having no contact with the grape skins. However, also for white grape varieties and wines, phenolic content is relevant. In fact, various modes of fermentation with skins have made their way into mainstream white winemaking for obtaining wines with interesting and distinct wine types. It is also known that the presence of skins during fermentation brings greater extraction of polyphenols in white wine; therefore, the study of these phenolic parameters presents a great importance as it contributes to the evaluation and application of new techniques of white wine production [5,6,7,8].

Since polyphenols are crucial for human health maintenance as well as playing an important role in the food quality assessment, their identification, determination, and quantification are essential aspects of modern analytical chemistry. More and more often, scientists are trying to develop better and better analytical procedures to determine polyphenols in different matrices [9,10].

Concerns and potential risks regarding the use of synthetic chemical antimicrobials and antioxidants have renewed the interests of consumers to use natural and safe alternatives [11]. Therefore, it is important to monitor the content of organic acids and polyphenols in wine samples, not only from the food (mainly wine) quality control point of view but also due to their beneficial potential properties to human health during light to moderate wine consumption.

During the ripening of grapes, sugar and flavonoids are accumulated, the content of organic acids decreases, and the concentration of volatile substances changes [12,13]. Apparently, a greater number of volatile compounds exist pre-veraison than post-veraison, as recorded for Riesling and Cabernet Sauvignon grapes, that also recorded differences (esters and aldehydes were the major class of compounds from Riesling grapes and alcohols for Cabernet Sauvignon) at veraison [12,13].

The composition of wine constituents, from a chemistry point of view, is a crucial aspect for the wine industry. Individual acids and oenological variables are as important as the sugar–acid balance in wine; therefore, detailed evaluation of all compounds present in wine is essential.

Wines consist of a diverse group of low- and high-molecular-weight compounds, including amino acids, organic acids, polyphenols, and carbohydrates, which makes the analysis a highly challenging task. Analysis of Polish wines has been reported since 2010. Wines of different regions of Poland have been analyzed, applying many varied techniques and determining diverse analytes, such as multiresidue pesticides [14,15], polyphenols [16,17], metals [18], and biogenic amines [19,20].

The use of microextraction techniques has had a very important boom in recent years. Techniques such as solid phase microextraction (SPME) with direct immersion (DI) [21], head-space (HS) [22] or fiber derivatization (FD) [23] modes, liquid phase microextraction (LPME) [24], dispersive liquid–liquid microextraction (DLLME) [25] are being accepted due to their low consumption of solvents and reagents and because they are considered environmentally friendly. In addition, these techniques could be easily coupled to gas chromatography (GC) and high-performance liquid chromatography (HPLC) [26].

Different microextraction techniques were used in the extraction and determination of different compounds in wine samples: Solid phase extraction (SPE) for resveratrol and other polyphenols [27], HS-SPME for wine esters [28], DLLME-GC for volatile phenols [29], and DLLME-HPLC for phenolic compounds [25]. In addition, ion pair dispersive liquid–liquid microextraction based on the solidification of a floating organic droplet (IP-DLLME-SFO) for phenolics acids [30]; SPME-FD for polyphenols [23]; and quick, easy, cheap, effective, rugged, and safe (QuEChERS) for multiclass polyphenols [31] are among the most important.

Porous membrane protected microsolid phase extraction (µ-SPE) was described by Basheer and co-workers in 2006. From that moment, more and more applications of this technique have been reported in different matrices and for different analytes. Among the evaluated performances, different groups of investigation assessed changes in type of sorbent, extraction conditions, and kind of analyzed samples [32,33,34,35,36].

A modification of µ-SPE was proposed by Sajid et al. (2019) who used the extraction devices filled with solid sample, instead of a sorbent material, reducing the steps involved in the process [37]. As an extension of those method criteria, a new technique involving ultrasound-assisted solvent extraction of porous membrane-packed liquid samples (UASE-PMLS) was optimized and validated in the presented work. Using the extraction device filled with liquid samples, extraction process is clean, fast, and easy to couple to GC–MS. Regarding other extraction techniques, the amount of solvent used is minimized and no waste is generated. Likewise, organic acids and polyphenols were evaluated in Polish wine samples by UASE-PMLS. Finally, to assess the green character of this new extraction technique, Analytical Eco-Scale and Green Analytical Procedure Index (GAPI) were used.

## 2. Results and Discussion

### 2.1. Optimization of an Ultrasound-Assisted Solvent Extraction of Porous Membrane-Packed Liquid Samples

Extraction solvent, extraction temperature, derivatization time, and sample pH were optimized. Since all of the evaluated factors were independent from each other on the obtained response and did not present interaction effects, they were performed as one variable at the time.

**Figure 1:**
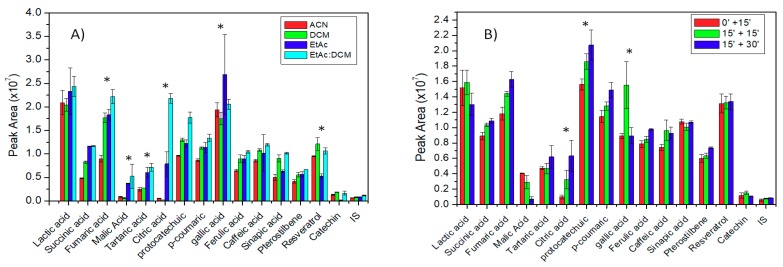
(**A**) Comparison of chromatographic responses in different extraction solvents. Error bars represent ±SD (*n* = 3). (**B**) Comparison of chromatographic responses at different derivatization times. Error bars represent ±SD (*n* = 3). The means differences are statistically significant (*p* < 0.05). (*): statistically different.

Behavior of compounds in each type of presented solvent significantly differed. Peak signal of some compounds showed bigger standard deviation in respect of other compounds in ethyl acetate (EtAc). Lactic and gallic acid showed this conduct. In addition, in ACN, some peaks in the chromatogram presented ghost peaks at different retention times. From six organic acids and nine polyphenols, all organic acids and seven polyphenols were successfully extracted with a good response in the EtAc/dichloromethane (DCM) (1:1) mix solvents (Figure 1A). Hence, the mixture of these low-polarity solvents was chosen for future experiments.

Regarding boiling points of the solvents used in the mixture (39.6 °C and 77.1 °C for DCM and EtAc, respectively), there was not much margin of proof without observing their transformation to the gas state and a scarce extraction of the analytes of interest. Therefore, the extraction process without additional temperature, with 25 °C and 40 °C in the ultrasonic bath, was evaluated, waiting for the temperature to cooperate with the flow of the analytes towards the extraction solvent. Without temperature, the maximum responses were obtained in four out of six organic acids and in eight out of nine polyphenols, while lactic acid, fumaric acid, and catechin showed their maximum signal with 25 °C applied. Thus, the following performances were realized without temperature application. This behavior could be observed because when temperature is applied to the extraction process, the solvent molecules compete between transformations to the gas state or analytes extraction.

Additionally, three times intervals of derivatization process were proposed. First, 15 min with the mixture of derivatizing agent and extraction solvent together; secondly, 15 min only with a derivatizing agent and another 15 min after adding the extraction solvent; and, finally, 30 min with derivatizing agent and 15 min additional after adding the extraction solvent to reconstitute the resulting derivatized compounds. The general trend observed was that at longer derivatization times, better responses were observed in most of the compounds (Figure 1B).

Although application of longer time could be observed a signal of greater intensity, the observed responses are not proportional to the times applied, so it can be assumed that longer time will not increase the responses significantly. In addition, too much time in the derivatization process would discriminate the application of this method for the routine work.

To understand the behavior of the analytes of interest, evaluation of different pH (1.30; 3.45; 4.80; 6.00) in the sample wine was also performed. Different behavior of examined compounds could be observed; the organic acid group is favored in strongly acidic medium (1.30) while the polyphenols group is benefited at less acidic medium pH 3.45. For this reason, it was a compromise to choose the pH 3.45 for the polyphenols analysis and acidic medium (pH 1.30) for the organic acids analysis in wine samples.

### 2.2. Method Validation

**Figure 2:**
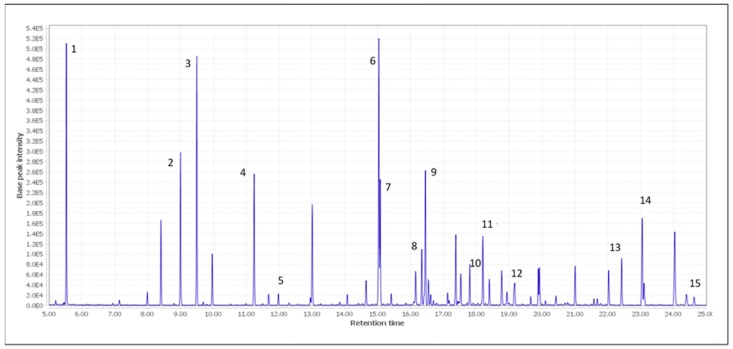
Chromatogram with identified compounds from standard solutions. 1. Lactic acid. 2. Succinic acid. 3. Fumaric acid. 4. L-Malic acid. 5. Tartaric acid. 6. Citric acid. 7. Protocatechuic acid. 8. p-Coumaric acid. 9. Gallic acid. 10. Ferulic acid. 11. Caffeic acid. 12. Sinapic acid. 13. Pterostilbene. 14. Resveratrol. 15. (+)-Catechin.

Selectivity was determined by the analysis of standard solutions, optimizing the conditions of separation and determination of the analytes of interest. Figure 2 shows a chromatogram with the determined 15 compounds. It can be seen that the compounds of interest were separated well, and the developed method could be used for the routine analysis of organic acids and polyphenols in wines.

Good linearity was obtained for the concentration ranges evaluated with *r* coefficient values between 0.9852 and 0.9993. Exact concentrations were expressed in the linear range, which corresponds to the calculation from the preparation of the standard solutions by weighing. Information about linear range, correlation coefficients, limit of detection (LOD), and limit of quantification (LOQ) is shown in Table 1. All parameters of method validation (intra- and inter-day precision and ME) were over 80% with coefficient of variation (CV) up to 17%. Recovery was between (92.0 ± 8.5)% and (113 ± 16)%. Results for each compound are shown in Table 2.

### 2.3. Quantification of Organic Acids and Polyphenols in Polish Wines

Fourteen out of 15 compounds were present in the wine samples (Table 3 and Table 4). Pterostilbene was not detectable and ferulic acid was not quantifiable in all samples. In red wines, succinic acid showed the maximum concentrations among organic acids. However, citric acid was the one registered at the lowest concentration (fumaric acid was not quantifiable in red wines). Among the polyphenols, (+)-Catechin was present at maximum level (>6000 mg/L) and sinapic acid at the minimum one.

In both rosé and white wines, L-Malic acid was the most abundant acid as predicted, while fumaric acid appeared at the lowest concentration level. In terms of polyphenols, the highest concentrations were noted for (+)-Catechin and caffeic acid in rosé and white samples, respectively. Sinapic acid was not quantifiable in white wines. The range of polyphenol concentrations (up to 40 µg/mL) was several orders of magnitude lower than organic acids (up to 4000 µg/mL), except for the (+)-Catechin in red wine.

**Figure 3:**
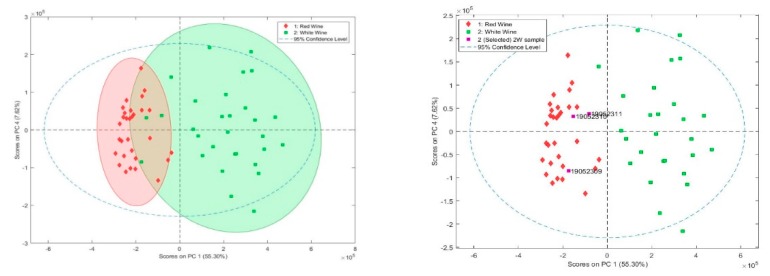
Principal component analysis (PCA) scores of the variables with PC1 and PC4 based on organic acids and polyphenols (retention times and *m/z* ions).

Data were explored using a principal component analysis (PCA), in order to investigate if it was possible to distinguish between white and red wine samples (rosé samples were not considered due to the too low number of samples). For this, PCA was performed with the entire data matrix from the information contained in the chromatograms (Rt and *m/z* ions). The PCA score graph resulting from the first and fourth major components (PC1 and PC4) is shown in Figure 3. Although there is an overlapping area of red (red diamonds) and white wine samples (green squares), a group of red and white wine samples are also observed, respectively, indicating that the discrimination between white and red wine samples was possible in principle. Samples that did not allow the best separation between red and white samples are the triplicate of 2W sample, in which organic acid concentrations were more similar to red wines than white wines. With the idea of obtaining a better classification of the wine samples, a linear discriminant analysis (LDA) was carried out in the next step.

In this case, rosé samples could not be used in the classification by LDA as the number of samples (three) was fewer than the number of variables (six for organic acids and seven for polyphenols). For the construction of the LDA graphs, the mean values of each compounds in the wine samples were used. Considering the analysis both in terms of organic acids and polyphenols, the discrimination between groups of samples was successful.

Regarding to organic acids, sample 2W differs in L-Malic and citric acids concentrations, being at a similar level to red wine samples (results similar to PCA). Despite that, a 95% percentage of classification could be observed. Regarding polyphenols, correct classification concerning white and red wines was 100%. This classification was achieved using the following seven variables: Protocatechuic acid, p-coumaric acid, gallic acid, caffeic acid, sinapic acid, resveratrol, and (+)-Catechin. This analysis presents evidence that the LDA tool allowed the determination of the variables with greater discriminant capacity.

With regard to polyphenols found in Polish wine, Raczkowska et al. (2012) [16] reported higher catechin and epicatechin concentrations and lower levels of resveratrol (<5 mg/L), results in line with those obtained in this work.

Concerning organic acids, similarly to the published results of Dobrowolska-Iwanek et al. (2014) [38], malic acid concentration in the examined white wines was higher than in the red wine. Malolactic fermentation and natural acid content of white grapes could explain these differences, while lactic acid showed an opposite behavior. There was significantly more lactic acid in red wines than in white wines. However, tartaric acid was detected at lower concentrations and with statistical differences between red and white wine samples (*p* = 0.017). These differences could correspond to the original concentrations of tartaric acid in the grapes used from the wines production.

If the results are compared with wines of different countries [2,39,40,41,42,43], one can observe high variances both for organic acids and for polyphenols. If the different grapes are grown in the countries characterized by different weather conditions, soil characteristics, and also viticultural practices, and the modifications in winemaking procedures are considered, these may be expected results. It would be unusual to expect a very strict concordance between the results obtained by other authors and those reported in the work developed here, since there are many variable conditions: Climate, vineyard, and chemical composition of the grape and soil; harvest; as well as the different winemaking techniques. The procedures with regard to isolation, maceration, and fermentation have an influence on the quality and composition of the final product, which may even differ from one batch to another [38].

### 2.4. Green Evaluation Assessment

With the growth of greener ideas, innovations in the minimization of techniques and reduction of solvent consumption and waste generation are taking place. Moreover, fewer and fewer derivatization processes are used. There is a general tendency to find appropriate techniques to determine analytes of interest without resorting to these processes that usually use large organic molecules as derivatizing agents. In this case, the derivation process could not be avoided. However, working with small volumes of reagents, only a few µL of these not so desired compounds was used. For this reason, both the technique applied here and some others reported in literature for organic acids and polyphenols determination were evaluated with available green assessment tools: Analytical Eco-Scale and GAPI index.

Based on the penalty points (PP) calculated for each procedure for organic acids determination (Table 5), one can notice that the highest score is achieved by procedure 2 (score: 94) based on water capillary ion analyzer. This signals that the following technique is the greenest in terms of being environmentally friendly, while just behind it, procedure 1 (this work) and procedure 4 are placed, with gathered scores of 88 and 87, respectively. Procedure 4 used electrospray ionization mass spectrometry (ESI-MS) while procedure 1 was based on GC–MS. The least green method, with a score of only 77, is procedure 3, where HPLC-UV was used.

In order to evaluate the greener conditions in polyphenols determination, Analytical Eco-Scale was also used (Table 5). In this case, one can observe the use of complex extraction techniques (liquid–liquid extraction (LLE) and microextraction by packed sorbent (MEPS)) and organic solvents (ACN or pyridine) were the more dangerous characteristics of these methods, obtaining low scores (all scores <90). Among the compared methods, procedure 1 (this work) presented the highest score (score: 88). Procedures 5 and 6 presented similar scores (83 and 84, respectively) and the least green method is procedure 7, which used LLE, an extraction technique that generates a high amount of waste and pyridine, a high toxic organic solvent, resulting in a score of 79.

**Figure 4:**
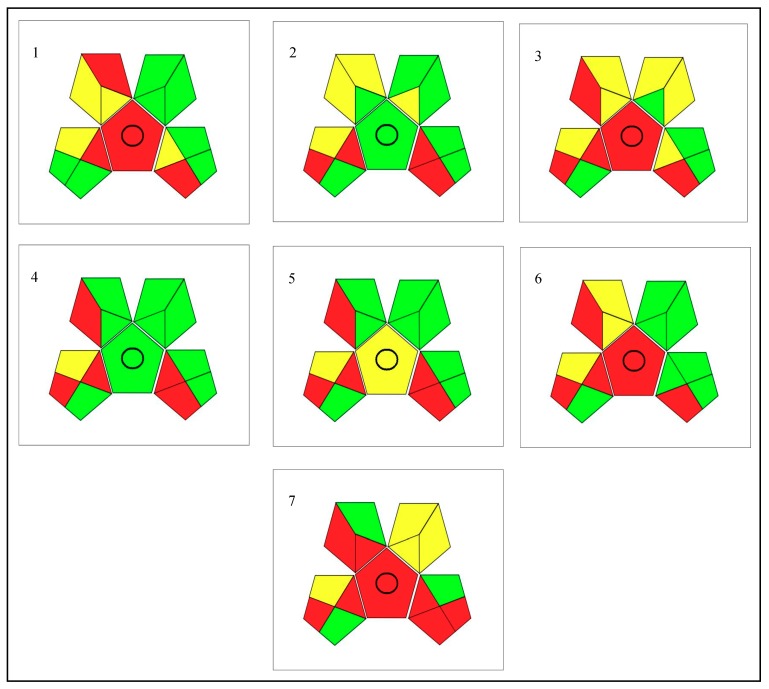
Assessment of the green profile of evaluated procedures for organic acid (2–4) and polyphenols (5–7) determination using Green Analytical Procedure Index (GAPI) tool. Procedure 1 is the one performed in this work.

Finally, GAPI tool [49] was applied to corroborate the amount of penalty points obtained. With this new tool, other variables that are included in the process are taken into account and are sometimes ignored in evaluations such as sample collection, preservation, transport, and storage; sample preparation; reagents, and compounds used; and instrumentation. In addition, the GAPI pictogram added a mark to identify if the method is for quantification or not. Figure 4 shows pictograms of GAPI index for all discussed procedures used in Analytical Eco-Scale evaluation.

It is easily visible that procedure 4 has the lowest and the least hazardous solvent and reagent used, which acts on its favor, placing given analytical practice before procedure 2 (best in Analytical Eco-Scale assessment).

Regarding the polyphenols, the red center represents the complex process due to extraction techniques (required in the most evaluated methods). To the naked eye, procedure 5 is the greenest among the evaluated methods, despite not having obtained the highest score on Analytical Eco-Scale.

## 3. Materials and Methods

### 3.1. Reagents

Lactic acid, succinic acid, fumaric acid, L-Malic acid, citric acid, tartaric acid, gallic acid, protocatechuic acid, caffeic acid, sinapic acid and (+)-Catechin were purchased from Sigma-Aldrich (St. Louis, MO, USA). The 3-methylbenzoic acid (internal standard, IS) was acquired from Sigma-Aldrich (St. Louis, MO, USA). p-Coumaric acid and ferulic acid were delivered from Fluka (Honeywell International Inc., North Carolina, USA). Resveratrol and pterostilbene were obtained from Extrasynthese (Extrasynthese, Genay, France). N,O-bis(trimethylsilyl)trifluoroacetamide (BSTFA) with 1% Trimethyl chlorosilane (TMCS) as derivatizing agent and HPLC-grade solvents (acetonitrile (ACN), dichloromethane (DCM), ethyl acetate (EtAc) and methanol) were delivered from Sigma-Aldrich (St.Louis, MO, USA). Polypropylene (PP) flat membrane sheet (Type PP 1E (R/P), pore size: 0.1 μm, wall thickness: 100 μm) was obtained from GVS Filter Technology (Roma, Italy).

### 3.2. GC–MS Conditions

Analyses were performed using a 7890A GC System (Agilent Technologies, Santa Clara, CA, USA) coupled to an Electron Ionization (EI) ion source and a 5975C single quadrupole MS (Agilent Technologies). A robotic autosampler and a split/splitless injection port were used. Injection port temperature was kept at 250 °C until the end of analysis. The separation of analytes was carried out on a Phenomenex ZB-5 MS capillary column (30 m × 0.25 mm internal diameter, and 0.25 μm film thickness, Shim-pol, Izabelin, Poland) with helium at a purity of 99.999% as the carrier gas in a constant flow of 1 mL/min. The methodology was based on a combination of parameters reported by Jurado Sanchez et al. (2011) [2] and Viñas et al. (2009) [23] with slight modifications. The oven temperature was programmed at 70 °C for 1 min, then increased to 280 °C at 10 °C/min and kept for 5 min. Total time was 27 min. The temperatures of the MS transfer line, ion source, and detector were set at 300, 230 and 150 °C, respectively. The MS was operated in positive mode (electron energy 70 eV). Full-scan acquisition was performed with the mass detection range set at *m*/*z* 40–600 to determine retention times of analytes, optimize oven temperature gradient, and to observe characteristic mass fragments for each compound. Data acquisition and analysis were executed by G1701EA GC/MDS Chemstation (version E.02.02.1431) (Agilent Technologies). For the identification and quantification of the analytes, single-ion monitoring (SIM) mode was used, with the ions listed in Table 6.

### 3.3. Wine Samples

Twenty-three bottles of wines from different regions of Poland were purchased in the Polish wine shop (Table 7). The wine bottles were protected from light and in consistent temperature (20 °C). The bottles of wine were opened a while before the analysis. Red wine (10 bottles), white wine (10 bottles) and rosé wine (three bottles) were analyzed.

### 3.4. Analytical Procedures and Statistical Analysis

#### 3.4.1. Extraction and Derivatization Procedure

**Figure 5:**
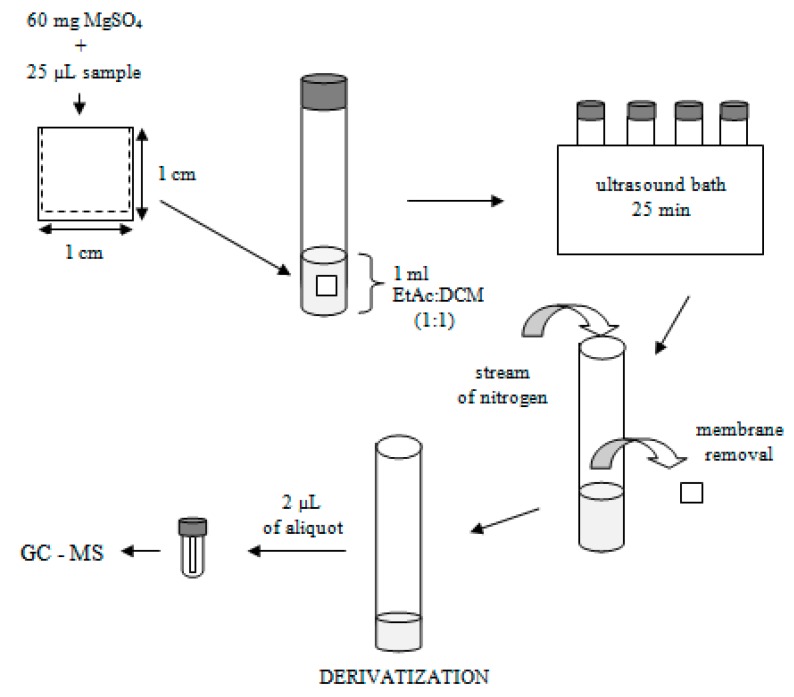
Scheme of the extraction procedure.

The extraction devices were made according to Basheer et al. (2007) [33]. One end of the membrane was kept open for filling of adsorbent and sample. A total of 60 mg of MgSO_4_ (support of liquid sample) and 25 μL of sample (spiked with standards of organic acids (OAs) and polyphenols or real sample wine) was filled, and the remaining end was heat-sealed (Figure 5). The dimensions of the extraction device were 1 cm × 1 cm. The extraction device was placed in a 4 mL glass vial with cap, and extraction solvent was added (1 mL to obtain the membrane completely submerged). The vial was immersed in an ultrasound bath and exposed for 25 min, allowing the extraction process to occur, according to Sajid et al. (2019) [37], at the optimal temperature. Then, the extraction device was removed from the vial with tweezers and discarded, and a stream of nitrogen was used to dry the extract. At that point, derivatization agent (N,O-bis(trimethylsilyl)trifluoroacetamide (BSTFA) with 1% trimethyl chlorosilane (TMCS)) was added (30 µL), and the vial was vortexed for 30 s and heated for 30 min (at 35 °C) to allow the derivatization process to occur. Then, an extraction solvent (170 μL) was added into the vial to reconstitute the derivatized analytes, and then they were heated for 15 min more at the same temperature. The subsequent solution was transferred to 200 μL insert placed in autosampler vials, and 2 μL aliquot was injected into GC–MS system for analysis. The optimization experiments and wine samples analysis were realized by triplicate. Efficiency extraction parameters were evaluated and optimized, including extraction solvent, extraction temperature, derivatization time, and sample pH. A comparison of chromatographic responses was used to evaluate the extraction efficiency.

#### 3.4.2. Quality Control (QC) Sample and Calibration Solutions

Analytes stock solution was prepared in methanol by diluting of analytical standards to reach a concentration of 1000 µg/mL. Then, the subsequent dilutions were prepared with MilliQ water (18.2 MΩ·cm). The stock solution of the IS was prepared also in methanol at a concentration of 100 μg/mL.

The standard solutions (*n* = 3) were prepared at approximately concentrations of 0.05, 0.1, 0.5, 1, 2.5, 5, 10, 15, 25, 50, 75, 100, and 150 µg/mL due to the wide levels found in wine samples. The concentration of the 3-methylbenzoic acid (IS) in each solution was maintained at 1 µg/mL. Calibration curves for organic acid were prepared at pH 1.30 and the polyphenols ones at pH 3.45.

Precision (intra- and inter-day) was evaluated with a QC sample at 10 µg/mL, prepared from the stock solution of analytes and the appropriate volume of IS followed by extraction procedure and GC–MS analysis (intra-day with *n* = 7 and inter-day with *n* = 5).

Spiked wine samples were prepared with 5 and 10 µg/mL for polyphenols (except gallic acid) and 15 and 20 µg/mL for organic acids and gallic acid to evaluate recovery. Recovery was expressed as % R ± U_%R_ (*k* = 2).

Matrix effect (ME) was performed in red, white, and rosé wine with spike samples with 5 µg/mL and expressed like accuracy (precision); precision calculated as coefficient of variation (CV).

#### 3.4.3. Method Validation

Standard validation parameters such as linearity, selectivity, sensitivity, limit of detection (LOD), limit of quantification (LOQ), and repeatability were evaluated. In addition, the recovery was calculated.

LOD was evaluated based on the regression parameters of the weighted calibration curves and was calculated using the following formula: LOD = 3.3 · S*_b_*/*a*, where S*_b_* is the standard deviation of the intercept and *a* is the slope of the calibration curve with the three lowest concentrations. LOQ was calculated as three times LOD.

#### 3.4.4. Statistical Analysis

Evaluation of the dataset of method validation was performed using the Excel files provided by Konieczka and Namiesnik (2018) [50]. Data from chromatograms were processed using MZmine and Matlab^®^ for the construction of principal component analysis (PCA). Linear discriminant analysis (LDA) was performed using The Unscrambler X.

## 4. Conclusions

Applying ultrasound-assisted solvent extraction of porous membrane-packed samples in liquid samples was a unique challenge due to a lack of previous reports in the scientific collected works. In this case, the use of sorbent (MgSO_4_) worked only like a support of liquid sample, but not like analytes sorbent. Analytes of interest passed through the membrane to extraction solvent, without another additional desorption step. Potential matrix interferences were retained inside the membrane. In addition, this method did not need a pre-conditioned treatment for the extraction device.

Among the most outstanding advantages from the different authors and proved in this work, the easiness in the extraction (due to the combination of cleanliness and concentration in one step), the miniaturization of the process, the minimization of the use of organic solvents (unwanted in the current analytical laboratories), and hence, limitation of generation of waste, stand out. In addition, the reduction of matrix effects was observed in complex samples, the application of the devices to semi-solid samples and, above all, the low cost involved in the process. Regarding the validation parameters, linearity, CV, and LODs of UASE-PMLS were acceptable and comparable with other techniques like SPE [34], SPME [32], and DLLME [51], among others. In addition, the recoveries were satisfactory. The main limitations of freshly developed extraction technique are related to the membrane bag, which cannot be reused as well as there is a risk of breakage during the extraction process.

Finally, the application to real samples was successful in all cases. The Analytical Eco-Scale and GAPI evaluations showed satisfactory results and underline its green performance, which means that the given technique can be considered environmentally friendly. Moreover, the developed extraction technique is a new tool that is expected to be explored for the determination of different analytes in diverse matrices, such as environmental and food samples, among others.

## Figures and Tables

**Table 1:** Quantification and calibration information for organic acids and polyphenols.

Compounds	Linear Range (µg/mL)	*r*	LOD (µg/mL)	LOQ (µg/mL)
Lactic acid	11–95	0.9927	3.6	11
Fumaric acid	0.83–30.63	0.9991	0.28	0.83
Succinic acid	5.6–25.4	0.9965	1.9	5.6
L-Malic acid	2.2–26.1	0.9882	0.75	2.2
Tartaric acid	25.13–150.75	0.9907	5.9	17
Citric acid	24–147.8	0.9852	8.2	24
Protocatechuic acid	1.1–32	0.9993	0.36	1.1
p-Coumaric acid	0.81–33.5	0.9981	0.27	0.81
Gallic acid	0.047–23.63	0.9915	0.016	0.047
Ferulic acid	1.0–32.5	0.9979	0.35	1.0
Caffeic acid	3.7–82.5	0.9992	1.2	3.7
Sinapic acid	0.95–30	0.9991	0.32	0.95
Pterostilbene	0.58–29	0.9989	0.17	0.52
Resveratrol	0.99–24	0.9983	0.33	0.99
(+)-Catechin	5.97–14.18	0.9942	2.0	5.9

LOD, limit of detection; LOQ, limit of quantification; *r*, correlation coefficient.

**Table 2:** Information about method validation and recovery for organic acids and polyphenols in Polish wines.

Compounds	Inter-day precision Accuracy (precision*) (*n* = 5) (*c* = 10 µg/mL)			Intra-day precision Accuracy (precision*) (*n* = 7) (*c* = 10 µg/mL)	Recovery		ME Accuracy (precision*) (*n* = 3) (*c* = 5 µg/mL)		
	Day 1	Day 2	Day 3		(*c* = µg/mL)	%R ± U_%R_(k = 2) (*n* = 5)	Red	White	Rosé
Lactic acid	95 (17)	101 (15)	102.8 (3.7)	99.7 (1.3)	15	111 ± 18	99.7 (9.3)	116.9 (8.0)	100.6 (1.4)
					20	109 ± 16			
Fumaric acid	103 (12)	98.4 (9.5)	101 (13)	109.1 (6.5)	15	104 ± 22	99.5 (2.6)	92.2 (1.2)	102.7 (4.8)
					20	92.0 ± 8.5			
Succinic acid	108.0 (9.4)	109.9 (2.6)	106 (12)	96.3 (4.9)	15	105 ± 15	94 (13)	94.9 (8.5)	98.7 (2.6)
					20	103.0 ± 7.8			
L- Malic acid	87.5 (6.3)	95 (15)	92.3 (6.9)	91.0 (5.5)	15	99.0 ± 4.4	102.2 (6.0)	103.26 (0.41)	99 (11)
					20	101.2 ± 8.9			
Tartaric acid	110 (14)	104 (16)	100.4 (8.2)	105.6 (1.2)	15	102.6 ± 5.7	87.0 (9.6)	102.8 (6.0)	95.1 (6.7)
					20	113 ± 16			
Citric acid	95 (13)	83 (15)	94.8 (9.8)	102.1 (7.7)	15	98.6 ± 3.8	110.7 (7.3)	107.4 (6.5)	111.9 (8.8)
					20	98.5 ± 3.1			
Protocatechuic acid	111.2 (7.7)	100.2 (6.4)	101 (12)	98.9 (7.2)	5	108 ± 15	107.7 (9.1)	112 (12)	108.7 (7.2)
					10	98.5 ± 3.6			
p-Coumaric acid	99.2 (8.2)	94.5 (6.1)	95.6 (9.0)	103.4 (7.0)	5	96 ± 15	96.0 (5.0)	107.9 (7.5)	90.3 (3.4)
					10	96 ± 12			
Gallic acid	109.5 (2.9)	102.5 (8.5)	99 (11)	104.2 (4.6)	15	94.5 ± 7.8	98.77 (6.8)	84.9 (2.2)	103.2 (4.3)
					20	98.7 ± 4.7			
Ferulic acid	93.1 (7.8)	91.3 (7.6)	92 (10)	99.4 (6.6)	5	103 ± 18	103.4 (6.9)	98.4 (2.6)	99.5 (2.2)
					10	102 ± 10			
Caffeic acid	94.9 (8.1)	100 (13)	117 (16)	95.0 (7.3)	5	101 ± 13	101.4 (3.6)	104.09 (0.58)	99.2 (2.5)
					10	93.7 ± 7.6			
Sinapic acid	103 (14)	99 (12)	109 (13)	104.9 (9.8)	5	106 ± 17	106.5 (4.1)	105.4 (8.5)	103.58 (0.29)
					10	103 ± 12			
Pterostilbene	103.9 (3.9)	110 (13)	112.8 (9.2)	100.7 (9.0)	5	102.3 ± 2.9	102.2 (5.0)	100.9 (2.0)	96.9 (3.0)
					10	110 ± 11			
Resveratrol	102 (12)	96 (15)	98.9 (8.5)	105.0 (7.5)	5	99 ± 16	114.7 (7.9)	99.6 (3.0)	96 (11)
					10	106 ± 13			
(+)-Catechin	98.0 (2.8)	107.9 (1.2)	106.5 (7.8)	108.3 (6.8)	5	95 ± 11	106.5 (2.3)	95.1 (4.8)	103.4 (7.2)
					10	97 ± 12			

* precision expressed as coefficient of variation (CV%).

**Table 3:** Concentration of polyphenols (µg/mL) in wine samples (*n* = 3) determined with the use of UAPM-LS GC–MS.

Sample	Protocatechuic Acid	p-Coumaric Acid	Gallic Acid	Ferulic Acid	Caffeic Acid	Sinapic Acid	Pterostilbene	Resveratrol	(+)-Catechin
1R	1.830 ± 0.027	7.82 ± 0.10	2.360 ± 0.077	<LOQ	25.18 ± 0.20	<LOQ	<LOD	2.880 ± 0.036	454 ± 112
2R	2.50 ± 0.10	10.62 ± 0.33	2.210 ± 0.081	<LOQ	15.41 ± 0.33	<LOQ	<LOD	2.460 ± 0.019	336 ± 61
3R	5.22 ± 0.45	2.990 ± 0.070	2.49 ± 0.51	<LOQ	9.14 ± 0.41	<LOQ	<LOD	2.960 ± 0.074	383 ± 36
4R	6.75 ± 0.72	11.65 ± 0.81	4.97 ± 0.91	<LOQ	21.03 ± 2.11	0.960 ± 0.035	<LOD	2.980 ± 0.060	965 ± 349
5R	2.48 ± 0.33	4.39 ± 0.61	2.51 ± 0.20	<LOQ	12.1 ± 1.4	<LOQ	<LOD	2.330 ± 0.052	66.9 ± 7.3
6R	<LOQ	12.33 ± 0.67	2.72 ± 0.16	<LOQ	25.3 ± 2.3	<LOQ	<LOD	2.540 ± 0.045	140 ± 19
7R	<LOQ	7.15 ± 0.79	6.59 ± 0.97	<LOQ	30.7 ± 3.0	<LOQ	<LOD	5.09 ± 0.23	6226 ± 243
8R	5.26 ± 0.12	10.87 ± 0.53	6.11 ± 0.41	<LOQ	8.36 ± 0.41	0.96 ± 0.41	<LOD	4.0 ± 0.14	2860 ± 116
9R	2.24 ± 0.17	7.12 ± 0.31	1.02000 ± 0.00051	<LOQ	14.3 ± 2.6	<LOQ	<LOD	2.280 ± 0.061	10.4 ± 2.9
10R	<LOQ	12.4 ±1.1	0.86 ± 0.51	<LOQ	10.2 ± 1.3	0.950 ± 0.041	<LOD	4.70 ± 0.54	5525 ± 994
1Ro	<LOQ	<LOQ	0.100 ± 0.012	<LOQ	6.52 ± 0.37	<LOQ	<LOD	2.320 ± 0.099	<LOQ
2Ro	2.37 ± 0.85	22.3 ± 3.8	1.18 ± 0.42	<LOQ	13.6 ± 3.2	1.03 ± 0.13	<LOD	2.290 ± 0.056	38 ± 13
3Ro	<LOQ	<LOQ	0.1200 ± 0.0006	<LOQ	5.49 ± 0.77	<LOQ	<LOD	2.210 ± 0.013	<LOQ
1W	<LOQ	1.110 ± 0.059	0.240 ± 0.014	<LOQ	8.88 ± 0.22	<LOQ	<LOD	2.2700 ± 0.0046	119 ± 12
2W	<LOQ	0.800 ± 0.052	0.190 ± 0.016	<LOQ	8.31 ± 0.15	<LOQ	<LOD	2.250 ± 0.021	41.9 ± 1.4
3W	<LOQ	0.94 ± 0.25	0.320 ± 0.051	<LOQ	6.17 ± 0.26	<LOQ	<LOD	2.270 ± 0.025	16.8 ± 4.7
4W	<LOQ	2.23 ± 0.33	0.470 ± 0.040	<LOQ	7.570 ± 0.040	<LOQ	<LOD	2.380 ± 0.029	34.9 ± 2.7
5W	<LOQ	0.9100 ± 0.0091	0.610 ± 0.093	<LOQ	7.69 ± 0.60	<LOQ	<LOD	2.360 ± 0.021	29.6 ± 1.5
6W	<LOQ	<LOQ	0.1200 ± 0.0072	<LOQ	5.78 ± 0.33	<LOQ	<LOD	2.2400 ± 0.0042	34.2 ± 6.4
7W	1.34 ± 0.21	1.23 ± 0.23	0.210 ± 0.013	<LOQ	6.33 ± 0.26	<LOQ	<LOD	2.510 ± 0.025	110 ± 28
8W	<LOQ	<LOQ	0.09 ± 0.10	<LOQ	4.4500 ± 0.0048	<LOQ	<LOD	2.2000 ± 0.0096	<LOQ
9W	<LOQ	1.1200 ± 0.0054	0.250 ± 0.020	<LOQ	5.27 ± 0.17	<LOQ	<LOD	2.250 ± 0.020	15 ± 11
10W	<LOQ	2.220 ± 0.042	0.300 ± 0.051	<LOQ	6.22 ± 0.12	<LOQ	<LOD	2.3100 ± 0.0043	29.9 ± 1.6

**Table 4:** Concentration of organic acids (µg/mL) in wine samples (*n* = 3) determined with the use of UAPM-LS-GC–MS.

Sample	Lactic Acid	Succinic Acid	Fumaric Acid	L-Malic Acid	Tartaric Acid	Citric Acid
1R	260.7 ± 4.5	259.3 ± 4.8	<LOQ	23.5 ± 1.8	58.9 ± 1.3	<LOQ
2R	340 ± 13	457 ± 11	<LOQ	117 ± 10	46.8 ± 3.5	<LOQ
3R	299 ± 18	456 ± 28	<LOQ	54.2 ± 3.6	44.8 ± 3.4	26.8 ± 3.4
4R	316 ± 37	465 ± 51	<LOQ	571 ± 62	48.5 ± 2.0	54 ± 17
5R	306 ± 23	466 ± 52	<LOQ	37.9 ± 4.8	65.0 ± 4.9	<LOQ
6R	316.0 ± 8.9	388 ± 28	<LOQ	31.1 ± 6.2	39.3 ± 2.8	<LOQ
7R	356.2 ± 1.9	351 ± 19	<LOQ	115 ± 29	39.9 ± 2.9	<LOQ
8R	333.3 ± 5.1	355.3 ± 2.4	<LOQ	217.7 ± 7.2	41.5 ± 1.6	32.9 ± 5.0
9R	310 ± 16	401.7 ± 1.4	<LOQ	20.9 ± 4.0	46.8 ± 4.5	<LOQ
10R	439 ± 60	370.7 ± 4.4	<LOQ	23.3 ± 3.2	43.3 ± 6.3	<LOQ
1Ro	125.6 ± 7.8	338 ± 34	<LOQ	867 ± 104	44.2 ± 4.1	163.33 ± 0.72
2Ro	221 ± 11	950 ± 287	1.38 ± 0.51	2185 ± 593	78.9 ± 7.9	371 ± 51
3Ro	320 ± 34	248 ± 13	<LOQ	205.7 ± 5.5	35.5 ± 1.5	187.6 ± 1.0
1W	45.2 ± 1.0	256 ± 12	<LOQ	870 ± 65	76 ± 11	163 ± 12
2W	328 ± 12	316 ± 31	<LOQ	175.4 ± 4.9	33.2 ± 2.3	<LOQ
3W	117.0 ± 3.9	310.9 ± 4.7	2.060 ± 0.087	901.7 ± 4.4	34.2 ± 2.9	206.2 ± 1.9
4W	53.9 ± 7.8	272 ± 29	1.55 ± 0.13	1081 ± 175	40.47 ± 0.80	222 ± 34
5W	79.6 ± 4.5	474 ± 16	3.10 ± 0.11	1421 ± 34	35.2 ± 1.3	329 ± 11
6W	60.5 ± 2.9	253 ± 18	<LOQ	921 ± 62	42.6 ± 2.4	172.6 ± 3.9
7W	99 ± 10	576 ± 34	1.47 ± 0.19	1158 ± 85	47.86 ± 0.51	312 ± 19
8W	75.9 ± 1.4	226 ± 19	<LOQ	1027 ± 61	33.1 ± 1.8	172 ± 22
9W	226 ± 16	338 ± 45	2.77 ± 0.36	1661 ± 247	37.5 ± 2.6	297 ± 33
10W	315 ± 19	385 ± 13	1.67 ± 0.11	1640 ± 87	34.5 ± 1.1	322 ± 35

**Table 5:** Calculated penalty points (PPs) for evaluated analytical procedures for organic acids (2–4) and polyphenols (5–7) determination in wine.

Procedure 1 (This Work)		Procedure 2 [44]		Procedure 3 [45]		Procedure 4 [46]		Procedure 5 [47]		Procedure 6 [48]		Procedure 7 [41]	
**Reagents**	**PPs**	**Reagents**	**PPs**	**Reagents**	**PPs**	**Reagents**	**PPs**	**Reagents**	**PPs**	**Reagents**	**PPs**	**Reagents**	**PPs**
**MgSO_4_**	0	NaH_2_PO_4_	0	benzylmalonic acid (IS)	1	Methanol	3	Formic acid	2	MeOH	3	Phenanthrene	1
**EtAc**	1	Na_2_HPO_4_	0	O-(4-nitrobenzyl)-N,N’-diisopropylisourea	2	Water	0	Acetonitrile	6	Formic acid	2	2,3-benzophenanthrene	3
**DCM**	1	Tetradecyltrimethyl ammonium hydroxide	2	Dioxane	6	Ammonium hydroxide	6	MeOH	3	Acetic acid	2	BSTFA + 1% TMCS	2
**MeOH**	3	CaCl_2_	0	Acetonitrile	6			Water	0	Ethanol absolute	3	Pyridine	3
**BSTFA + 1% TMCS**	2	Water	0	Water	0					NaOH	2	EtAc	1
**3-methylbenzoic acid (IS)**	0									Water	0		
	Ʃ 7		Ʃ 2		Ʃ 15		Ʃ 9		Ʃ 11		Ʃ 12		Ʃ 10
**Instruments**	**PPs**	**Instruments**	**PPs**	**Instruments**	**PPs**	**Instruments**	**PPs**	**Instruments**	**PPs**	**Instruments**	**PPs**	**Instruments**	**PPs**
**Transport**	1	Transport	1	Transport	1	Transport	1	Transport	1	Transport	1	Transport	1
**GC–MS**	2	Water Capillary Ion Analyzer	2	Heater	2	ESI-MS	2	Filtration	0	MEPS	2	LLE	2
**Ultrasound bath**	1	Occupational hazard	0	HPLC-UV	2	Occupational hazard	0	UFLC-MS/MS	2	UHPLC-PDA	0	GC–MS	2
**Occupational hazard**	0	Waste	1	Occupational hazard	0	Waste	1	Occupational hazard	0	Occupational hazard	0	Occupational hazard	1
**Waste**	1			Waste	3			Waste	3	Waste	1	Waste	5
	Ʃ 5		Ʃ 4		Ʃ 8		Ʃ 4		Ʃ 6		Ʃ 4		Ʃ 11
**Total PPs: 12**		Total PPs: 6		Total PPs: 23		Total PPs: 13		Total PPs: 17		Total PPs: 16		Total PPs: 21	
**Score: 88**		Score: 94		Score: 77		Score: 87		Score: 83		Score: 84		Score: 79	

**Table 6:** List of determined compounds, their retention times, and selected ions for single-ion monitoring (SIM) mode.

Labeled Peaks	Compound	Rt [min]	Quantitative Ion	Qualitative Ions
1	Lactic acid	5.50	147	117, 191
2	Succinic acid	9.01	147	75, 148
3	Fumaric acid	9.50	245	147, 246
4	L-Malic acid	11.24	73	147, 233
5	Tartaric acid	11.98	73	147, 292
6	Citric acid	15.04	273	147, 347
7	Protocatechuic acid	15.08	193	355, 370
8	p-Coumaric acid	16.35	293	219, 308
9	Gallic acid	16.45	281	282, 458
10	Ferulic acid	17.80	338	308, 323
11	Caffeic acid	18.20	219	396, 397
12	Sinapic acid	19.16	368	353, 338
13	Pterostilbene	22.42	328	327, 329
14	Resveratrol	23.05	444	445, 446
15	(+)-Catechin	24.63	368	355, 369
16	IS	9.55	193	119, 149

**Table 7:** Wine sample characteristics.

Label	Vineyard	Year	Type of Wine	Origin	% Alcohol	Grape Type	Sugar Content
1R	HOPLE Winnica Poraj Paczkow	2015	Red	Paczkow	11.0	Regent	dry
2R	Winnica Chodorowa	2017	Red	Grybów	12.0	Regent	dry
3R	Dom Bliskowice	2014	Red	Bliskowice	12.1	Rondo	dry
4R	Winnica Witanowice	2013	Red	Witanowice	12.5	Regent	dry
5R	Adoria Vineyards	2017	Red	Zachowice	13.5	Dornfelder	dry
6R	Winnica Chodorowa	2017	Red	Grybów	11.0	Rondo	dry
7R	Adoria Vineyards	2017	Red	Zachowice	13.5	Pinot Noir	dry
8R	Winnica Turnau	2016	Red	Banie	13.0	Rondo/Regent	dry
9R	HOPLE Winnica Poraj Paczkow	2015	Red	Paczków	11.5	Rondo	dry
10R	Winnica Golesz	2016	Red	Jasło	12.5	Mix of three grapes	dry
1W	Winnica Solaris	2016	White	Opole Lubelskie	12.0	Johanniter	dry
2W	Adoria Vineyards	2017	White	Zachowice	12.0	Riesling	semi-dry
3W	Winnica Saint Vincent	2016	White	Borów Wielki	12.0	Pinot Gris, Riesling, Muscat Ottonel, Gewurztraminer	semi-dry
4W	Winnica Srebrna Gora	2017	White	Kraków	12.0	Seyval Blanc, Hibernal, Johanniter, Solaris	semi-dry
5W	Winnica Saint Vincent	2016	White	Borów Wielki	13.0	Pinot Gris	semi-dry
6W	Winnica Solaris	2016	White	Opole Lubelskie	12.5	Solaris	sweet
7W	Winnica Witanowice	2014	White	Witanowice	12.0	Bianca	dry
8W	Winnica Turnau	2017	White	Banie	12.5	Solaris	dry
9W	Winnica Golesz	2017	White	Jasło	12.0	Mix of grapes	semi-sweet
10W	Winnica Golesz	2015	White	Jaslo	11.5	Mix of eight grapes	dry
1Ro	Winnica Srebrna Gora	2014	Rosé	Krakow	10.5	Zweiglet	semi-dry
2Ro	Winnica De Sas	2015	Rosé	Krosnice	10.5	Regent	dry
3Ro	Winnica Golesz	2016	Rosé	Jaslo	11.5	Mix of three grapes	dry
